# Microplastics Reduce Short-Term Effects of Environmental Contaminants. Part I: Effects of Bisphenol A on Freshwater Zooplankton Are Lower in Presence of Polyamide Particles

**DOI:** 10.3390/ijerph15020280

**Published:** 2018-02-06

**Authors:** Saskia Rehse, Werner Kloas, Christiane Zarfl

**Affiliations:** 1 Leibniz-Institute of Freshwater Ecology and Inland Fisheries, Müggelseedamm 310, D-12587 Berlin, Germany; werner.kloas@igb-berlin.de; 2 Center for Applied Geosciences, Eberhard Karls Universität Tübingen, Hölderlinstraße 12, D-72074 Tübingen, Germany; christiane.zarfl@uni-tuebingen.de; 3 Department of Endocrinology, Institute of Biology, Humboldt-Universität Berlin, Invalidenstraße 110, D-10115 Berlin, Germany

**Keywords:** microplastics, vector effect, bisphenol A, polyamide, freshwater zooplankton, acute toxicity

## Abstract

Microplastics can have direct physical effects on organisms in freshwater systems, and are considered as vectors for absorbed environmental pollutants. It is still under discussion if microplastics are relevant pollutant vectors for uptake into aquatic organisms in comparison to further uptake pathways, e.g., via water or sediment particles. We analyzed how the presence of microplastics (polyamide particles, PA) modifies acute effects of the environmental pollutant bisphenol A (BPA) on freshwater zooplankton (*Daphnia magna*). Daphnids were exposed to PA particles and BPA alone, before combining them in the next step with one concentration of PA and varying concentrations of BPA. The PA particles themselves did not induce negative effects, while the effects of BPA alone followed a typical dose-dependent manner. Sorption of BPA to PA particles prior to exposure led to a reduction of BPA in the aqueous phase. The combination of BPA and PA led to decreased immobilization, although PA particles loaded with BPA were ingested by the daphnids. Calculations based on physiochemistry and equilibrium assumptions indicated lower BPA body burden of daphnids in the presence of PA particles. These results confirm model-based studies, and show that investigated microplastic concentrations are negligible for the overall pollutant uptake of daphnids with water as additional uptake pathway.

## 1. Introduction

The presence of different stressors, like pollutants and their interactions, leads to complex scenarios in the environment. Freshwater systems are not only polluted by chemical substances, but also by microplastics (plastic particles < 5 mm, [1]). Microplastics have been considered as potentially harmful to freshwater organisms (e.g., [2] for an overview). In particular, concurrent effects of microplastics and other pollutants are challenging to evaluate, because of diverse causalities under environmental conditions [3]. Therefore, it is important to systematically identify not only effects of microplastic material itself, but also interactions of microplastics with other pollutants, and their potential combined effects on freshwater organisms.

In the past, rivers have been considered as a source for marine litter, including plastics [4]. More recent studies confirmed that rivers and lakes are ubiquitously polluted with microplastics [5,6,7,8]. Besides the high variety of characteristics of microplastics, some sizes, shapes, and polymer types are reported to be more abundant in freshwater. Irregular shaped plastic fragments and very small microplastics, in the range of only a few to some hundreds of micrometers, make up a big proportion of the observed overall amount of microplastics in surface waters and beach sediments [8,9,10]. Polyamide (PA) and polyethylene (PE) are among the most abundant polymer types found in environmental samples. Due to limits in sampling and evaluation techniques, especially data on concentrations of microplastics in the range of a few micrometers, and microplastics in the free water zone are still scarce, and sometimes not directly comparable, because of different reference units. Results are often either given as number of microplastic particles or mass of microplastics per area (e.g., m^2^) or per volume (e.g., m^3^). Monitoring so far has shown that mass concentrations of microplastics in surface waters range from 10^−3^ to 10^−1^ mg m^−3^ for rivers, and from 10^−3^ to 10^−1^ mg m^−2^ for lakes [8,11]. Particle numbers of microplastics in both rivers and lakes, go up to 10 particles m^−3^ in samples from surface water [8,11,12]. Asian rivers seem to be the most polluted, with up to 1000 microplastic particles m^−3^ and 1000 mg m^−3^ in Yangtze river [13,14].

Until now, the majority of studies about the potential harm of organisms by microplastics focused on marine species (e.g., [15] for an overview). First results on freshwater organisms showed that microplastics may harm these in a similar way like they do with marine organisms, especially species of similar functional groups or with comparable food acquisition strategies, e.g., filtering organisms [2,16]. By filtering surrounding water, organisms like zooplankton or mussels are prone to water contaminants, in particular. Daphnids play an important role in lake ecosystems at the base of the food web as effective consumers of algae and bacteria, and are an important prey for, e.g., fish larvae. Similarly to bigger sized plastics, raw microplastic material can have negative impacts on freshwater organisms by itself (physical effects), especially after ingestion [17,18,19,20]. However, only high concentrations of some microplastic types induced negative effects in laboratory experiments. Effects range from acute effects, like increased mortality in amphipods and daphnids, and immobilization in daphnids, to chronic effects like decreased growth in algae and lower reproduction rates in amphipods. If small enough, microplastics can translocate within the body and enter tissues, as shown for 1 μm polystyrene (PS) particles in oil storage droplets of daphnids [21]. Being some orders of magnitude smaller than microplastics, nanoplastics were shown to have a bigger negative impact on aquatic organisms than microplastics [22].

For the risk assessment of microplastics, not only physical effects but also interactions with organic pollutants need to be considered (chemical effects). Hydrophobic organic pollutants (HOC) are of special concern for freshwater ecosystems [23]. Being lipophilic, they tend to sorb to natural organic material like sediments, and to bioaccumulate in aquatic organisms. Some organic pollutants are associated with the production of plastics. Various chemicals are used as starting material for polymerization (e.g., bisphenol A, BPA), or as additives for adjusting properties of the polymer depending on its intended application (e.g., colorants, plasticizers, UV stabilizers). Similar to natural organic material, microplastics tend to sorb organic pollutants from the water column, but also leach chemicals used for manufacturing [24]. This is why microplastics are considered as vectors for pollutants to aquatic organisms in general, especially if microplastics are ingested [25]. Due to the high affinity of HOC to plastics, microplastics are considered as vectors for HOC in particular [26,27]. BPA is used for the production of polymer types like polycarbonates and epoxy resins, and was shown to sorb into microplastics in freshwater with mean concentrations of 16.6 ng g^−1^ [8]. It is hormonally active in freshwater vertebrates, with disruption of larval development and the thyroid system in amphibians [28,29]. This is why BPA is classified as an endocrine disruptor. In freshwater zooplankton, BPA induces moderate acute toxicity (EC_50_ after 48 h of 10 mg L^−1^, [30]). BPA has been shown to leach from products used in households, including products associated with food consumption, and is considered as a potential direct threat to humans [31].

That microplastics can act as vector for organic pollutants and modulate effects of pollutants was demonstrated in laboratory feeding experiments with fish [32,33,34]. Trophic transfer of microplastics and sorbed pollutants was shown in zebrafish [35]. While this identifies microplastics as a potential source for pollutants in general, the relative importance of microplastics as vector is not clarified. After exposure of bivalves with environmentally relevant concentrations of microplastics spiked with polychlorinated biphenyls (PCBs), no PCBs could be detected in the bivalves, and in fish which were feeding on the bivalves [36]. Model-based studies indicate that the vector function of microplastics is negligible in the environment compared to other uptake pathways and that sorption equilibrium can be assumed for most marine microplastics on relevant timescales [3,37,38]. This is also supported by a modelling study showing that leaching of additives from microplastics is not a relevant exposure pathway for lugworms [39]. Validation by empirical data is needed to raise credibility for this evidence. On a microorganism level, Kleinteich et al. show that the effect of polycyclic aromatic hydrocarbons on bacterial community composition was reduced in the presence of microplastics ([40], this issue). Recent studies with marine organisms indicate no or only low impact of microplastics as carrier for pollutants [38,41,42,43]. Due to similar modes of action, this highlights the need for studies with experimental evidence in freshwater organisms.

The underlying hypothesis of this study is that microplastic particles do not increase, but rather reduce the effect of a pollutant that is already available in the aqueous phase. Assuming a system in equilibrium, sorption of the contaminant to microplastics leads to removal from the aqueous, i.e., bioavailable, fraction. To test these hypotheses freshwater zooplankton (*Daphnia magna*) was exposed to BPA and PA particles as model compounds in equilibrium batch systems. Immobilization was analyzed as criterion for negative effects for (1) PA particles alone, (2) BPA alone, and (3) BPA in presence of PA particles. Results of this approach aim to entangle the discussion on the potential vector effect of microplastics for environmental pollutants in freshwater systems.

## 2. Materials and Methods

### 2.1. Microplastic Material and Chemicals

Polyamide particles (PA) were purchased as powder from Goodfellow (Nylon 6, AM306010; Goodfellow GmbH, Bad Nauheim, Germany). The particles had an irregular shape, a mean diameter of 15–20 μm (min. 5 μm, max. 50 μm) and a polymer material density of 1.13 g cm^−3^. Bisphenol A (BPA, ≥99%, CAS number 80-05-7) was purchased from Sigma-Aldrich (Sigma-Aldrich Chemie GmbH, Munich, Germany). Stock solutions of BPA with 40 mg L^−1^ were prepared with freshly prepared artificial daphnia culture medium (ADaM medium, [44]), and stored for a maximum of two days at 7 °C. Glass material was used for, e.g., preparation of test solutions whenever possible.

### 2.2. Study Design

To test our hypotheses, the experimental setup for the exposure of daphnids needed to meet several predefined conditions. For analyzing the potential vector effect of microplastics, the sorption of a quantifiable proportion of BPA to the PA particles, and the uptake of the particles by the daphnids, in general, needed to be assured. Sorption and desorption processes of BPA on PA particles needed to be in an equilibrium state. Finally, the particles themselves should cause neither chemical (e.g., by leaching additives) nor physical acute effects on daphnids, that could confound effects of the pollutant itself.

#### 2.2.1. Sorption Characteristics and BPA Content of PA Particles

To analyze sorption characteristics of BPA to PA particles and determine when sorption equilibrium is reached, a batch experiment was performed with one concentration of BPA (10 mg L^−1^) and PA particles (microplastics, MP; 200 mg L^−1^). Mixtures contained BPA together with PA particles (BPA + MP), and were compared to mixtures with only BPA as control (BPA alone). A batch with only PA particles (MP alone) was analyzed in addition, to make sure that no BPA was leaching out of the PA particles themselves. For treatments containing PA particles (BPA + MP, MP alone), 50 mg of PA particles were weighed into 20 mL glass flasks, rinsed with either BPA solution (BPA + MP) or ADaM medium (MP alone), and mixed thoroughly. The mixtures were decanted to 500 mL glass bottles, which were then filled up to a total volume of 250 mL with BPA solution (BPA + MP, BPA alone) or ADaM medium (MP alone), and shaken at 200 rpm. The concentration of BPA dissolved in water was measured regularly for up to 72 h after removing the PA particles with a syringe filter via high performance liquid chromatography (HPLC). For BPA in combination with microplastics, the concentration of BPA decreased with reaching equilibrium, after 48 h at 7.5 mg L^−1^. In batches with microplastics alone, no BPA was detected above the detection limit of 0.1 mg L^−1^ (for more detailed information on methods and results, see Supplementary Materials). Sorption of BPA to glass surfaces and degradation of BPA were assumed to be negligible, because of good recovery rates in batches with only BPA.

Assuming equilibrium after 48 h, the partition coefficient, defined as [45]

$$K_{pa,w}=\frac{c_{pa}}{c_{w}}$$

was calculated with $c_{pa}$ as equilibrium concentration of BPA adsorbed to PA particles (in mg kg^−1^), and $c_{w}$ as equilibrium concentration of BPA in water measured by HPLC (7.5 mg L^−1^, Table S1). $c_{pa}$ was calculated from the BPA mass balance as follows:

$$c_{pa}=\frac{m_{total}-c_{w}*V_{w}}{M_{pa}}$$

with $m_{total}$ as total mass (in mg) of BPA in the system, $V_{w}$ as volume of water in the beakers (in L), and $M_{pa}$ as mass of PA particles (in kg) added to the beakers.

#### 2.2.2. Pre-Exposure with Single Substances

A clone of *D. magna* (originally isolated from Großer Binnensee, [46]) from a healthy laboratory stock was cultured according to Rehse et al. [19]. Potential uptake and effects of pristine PA particles alone were studied by exposing daphnids not older than 24 h (neonates) to a broad range of concentrations of PA particles (25–250 mg L^−1^). The particles were dispersed in the water column at the beginning of the exposure, but settled at the bottom of the test beakers shortly after. Ingestion of particles not only from the water column, but also from settled material, was observed within the first 24 h [47,48]. Ingestion of particulate matter as potential food for daphnids is size-dependent, with an optimum range between 0.7–70 μm in diameters [49,50]. Daphnids are unselective filter feeders, so ingestion of PA particles with a size range between 5–50 μm in diameter could be expected. No daphnids were immobilized after 24 or 48 h of exposure to PA particles at any concentration (25–250 mg L^−1^). Therefore, both physical and chemical effects i.e., by leaching additives, can be excluded.

For finding the range of BPA concentrations relevant for acute toxicity, daphnids were also exposed to a broad concentration range of BPA alone (2.5–40 mg L^−1^). The concentration at which 50% of the daphnids were immobilized (effective concentration, EC_50_) was calculated as benchmark. If the EC_50_ value is lower for one treatment, daphnids are assumed to be more sensitive towards the tested pollutant. The EC_50_ of 7.6 mg L^−1^ after 48 h is similar to others reported in the literature [51], suggesting similar or slightly higher sensitivity of our daphnid stock.

### 2.3. Exposure Experiments with Mixtures of BPA and PA Particles

To analyze how the presence of PA particles modulates the effects of BPA, daphnids were exposed to treatments with five different initial nominal concentrations of BPA (5, 7.5, 10, 12.5, and 15 mg L^−1^). Each concentration of BPA was tested alone (BPA alone) and with PA particles (BPA + MP) with a constant concentration of PA particles (200 mg L^−1^), leading to five pairs of treatment combinations.

Test solutions were prepared as described for the pre-experiment on sorption characteristics of PA particles (see Supplementary Materials). One control treatment contained ADaM medium only. All test solutions were shaken in glass bottles as batches for 48 h prior to exposure experiments, to ensure sorption equilibrium. BPA concentrations in the water of batches were checked after 0, 24, and 48 h of shaking via HPLC, to validate sorption equilibrium. Each test beaker was then filled with 40 mL of test solution. A total of 25 neonates in groups of five animals were exposed to each treatment (*n* = 5 per treatment) following the *Daphnia* sp. Acute Immobilization Test, for full assessment of acute toxicity [52]. According to the guideline, immobilization after 24 and 48 h was the criterion for negative effects. If daphnids were not able to swim within 15 s after gentle agitation of the test vessel, individuals were considered to be immobile. Daphnids were not fed during exposure. Temperature, pH, and oxygen were measured in an extra beaker without daphnids, with one beaker for each treatment and processed the same way. Measurements were all in the same range after 24 and 48 h of exposure (22.6 °C, pH 7.5, 8.6 mg O_2_ L^−1^). Concentrations of BPA dissolved in water were measured via HPLC in the test beakers at the beginning (0 h) and at the end of exposure (48 h). The mass balance of BPA was used based on physiochemical characteristics to analyze the distribution of BPA within the different compartments (water, PA particles, organisms) and determine the theoretical BPA concentration in water $c_{w}$ in both experimental setups, i.e., for BPA alone:

$$c_{w}=\frac{m_{total}}{V_{w}+BCF*M_{org}}$$

and for BPA in combination with microplastic particles:

$$c_{w}=\frac{m_{total}}{V_{w}+BCF*M_{org}+K_{pa,w}*M_{pa}}$$

with $m_{total}$ as total mass (in mg) of BPA in the system, $V_{w}$ as volume of water in the beakers (in L), the bioconcentration factor (BCF), i.e., the partition coefficient between the organic phase (organisms) and water (in L kg^−1^), $K_{pa,w}$ as partition coefficient between PA particles and water (in L kg^−1^), and $M_{\mathrm{pa}}$. as mass of PA particles (in kg). BCF was calculated according to Veith et al. [53], leading to 225.95 L kg^−1^. The partition coefficient of $K_{pa,w}=1666\;{L\;\mathrm{kg}}^{-1}$ was calculated from pre-experiments on sorption characteristics.

Measured concentrations of BPA in water in the test beakers before exposure were compared to calculated concentrations. EC_50_ values were calculated with immobilization rates and measured BPA concentrations after 48 h of exposure for BPA alone and BPA in combination with microplastics.

### 2.4. Statistical Analysis

Treatments with BPA alone and BPA in combination with microplastics were tested for significant differences with two-sided Fisher’s exact test with the software GraphPad Prism (version 4.03, GraphPad Software, Inc., San Diego, CA, USA) in pairwise comparison for nominal BPA concentrations. The EPA trimmed Spearman–Karber (TSK) program (version 1.5; U.S. Environmental Protection Agency, Washington, DC, USA) was used for calculating EC_50_ values, including 95% upper (UC) and lower (LC) confidence intervals.

## 3. Results

### Exposure Experiments with Mixtures of BPA and PA Particles

**Figure 1:**
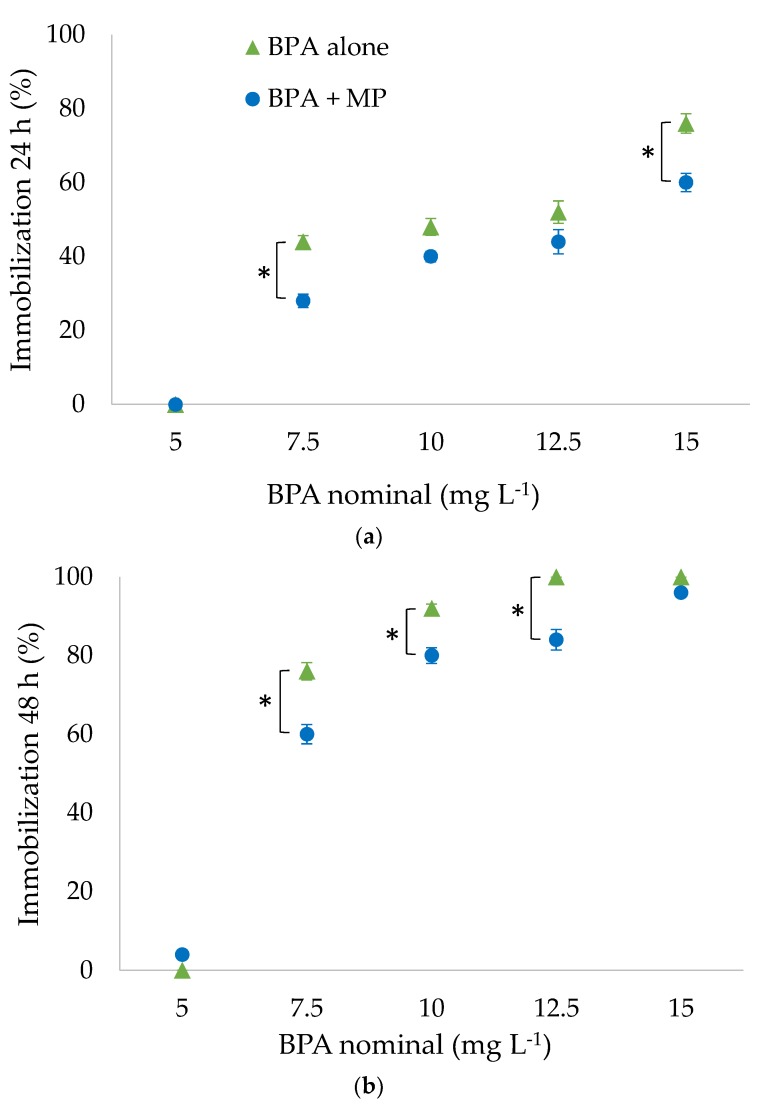
Immobilization of daphnids after (**a**) 24 h; and (**b**) 48 h of exposure with bisphenol A (BPA) and microplastics (MP), in different treatments with increasing nominal BPA concentrations for BPA alone and BPA in combination with microplastics (BPA + MP; 5–15 mg L^−1^), and one concentration of microplastics for BPA + MP (200 mg L^−1^; mean ± SE, *n* = 5). Brackets marked with asterisks indicate significant differences (Fisher’s exact test, *p* < 0.05) between treatments with BPA alone and BPA + MP.

We observed that the intestines of the daphnids appeared to be whitish after 24 h already in all treatments with BPA combined with microplastics, indicating ingestion of particles. Being equally distributed within the water column at the beginning of exposure, the particles sank to the bottom of the test beakers, forming a thin layer within the first 24 h. Immobilization rates of daphnids after 24 and 48 h increased, following the gradient of the nominal BPA concentration (Figure 1). Immobilization after 48 h of exposure increased in comparison to 24 h. Treatments with BPA in combination with microplastics always caused reduced immobilization compared to BPA alone. After 24 and 48 h, some treatments differed significantly when directly comparing treatments with the same nominal BPA concentration in the presence (BPA + MP) and absence of particles (BPA alone). The concentrations of BPA in water, measured by HPLC, were lower for BPA in combination with microplastics, compared to BPA alone, during exposure of daphnids (Table S2). Concentrations in water were stable until the end of exposure after 48 h, with only a small decrease of BPA, which could be due to degradation of only a small proportion of BPA in the presence of daphnids.

**Figure 2:**
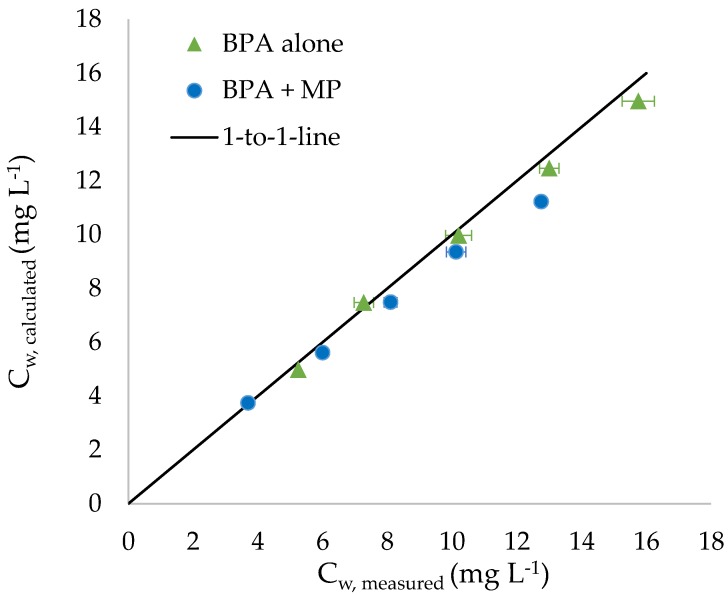
Relationship between measured (means ± SD, *n* = 5) and calculated concentrations of bisphenol A (BPA) in water for treatments with BPA alone and BPA combined with microplastics (MP) (treatment BPA + MP).

Calculated BPA concentrations in water ($c_{w,calculated}$) are close to measured values ($c_{w,measured}$), confirming accuracy of the measurements and that sorption equilibrium was reached (Figure 2).

According to the calculations, sorption of BPA to microplastics led to a reduction of nearly 25% of BPA in water, in BPA combined with microplastic treatments, compared to BPA alone (Table 1). Taking 0.28% of BPA in daphnids from BPA alone as reference, daphnids in BPA combined with microplastics hold 25%.

**Figure 3:**
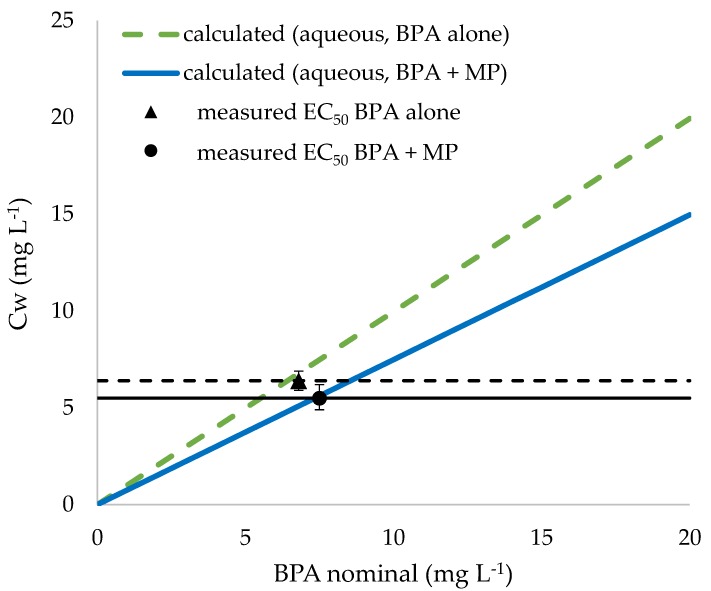
Calculated concentrations of bisphenol A (BPA) in water for BPA alone and BPA in combination with microplastics (MP) (treatment BPA + MP) represented as colored lines, together with EC_50_ values based on measured concentrations of BPA in water and corresponding EC_50_ lines.

EC_50_ based on measured BPA concentrations in water is lower for BPA in combination with microplastics (BPA + MP; 5.54, LC: 4.98, UC: 6.15 in mg L^−1^) than for BPA alone (6.4, LC: 5.94, UC: 6.87 in mg L^−1^; Figure 3). Overlap of confidence intervals indicates no significant differences between the EC_50_ values.

## 4. Discussion

In contrast to studies that assign microplastics an important role as pollutant vector, this experimental study found no evidence for this. Having the same total mass of BPA in the system, even fewer daphnids were immobilized in the presence of PA particles than without. This was despite that clear evidence could be provided for sorption of BPA to the particles and ingestion of the PA particles by the daphnids.

### 4.1. Study Design with Predefined Conditions

Recently, there was a call for more complex experimental setups with scenarios likely encountered in the environment for the risk assessment of microplastics [3,38]. Including only selected predefined parameters under well controlled laboratory conditions in the setup of this study provided the opportunity to focus on a more mechanistic understanding of single relevant aspects. In many previous studies, the potential vector effect of microplastics was analyzed by measuring uptake rates of the pollutants assessed by tissue concentrations as proxy [22,54]. Other studies analyzed pollutant effects, e.g., biomarker activity or histopathological changes [42,55]. This study focused on analyzing how the acute effects of a pollutant are modified by microplastics, rather than measuring uptake rates and tissue concentrations of the pollutant within the daphnids. Experimental results on immobilization in daphnids and analytical measurements of BPA in water were complemented by calculations of the mass distribution of BPA in the test system.

Sorption behavior of pollutants to microplastics is crucial for their potential vector effect. Batch experiments showed that PA particles are an intermediate strong sorbent for BPA with fast sorption equilibration. Shaking the mixtures for 48 h prior to testing assured that sorption processes of BPA to PA particles were in an equilibrium state. In most previous studies, microplastics loaded with organic pollutants were directly fed to the test animals without reaching sorption equilibrium in the test system before exposure [34,43]. Even if microplastic pollutant mixtures have been pre-equilibrated prior to exposure to ensure sorption of the pollutant to microplastics in some studies, dilution of the mixtures led to a non-equilibrium state at the beginning of exposure [32,42,54]. While this non-equilibrium state is also a relevant environmental scenario with contaminated microplastics emitted into the aqueous system, e.g., via point sources, sorption equilibrium for microplastics and pollutants can be expected for microplastics being in the environment for a longer time [3], and allows estimation of the contaminant distribution within the experimental setup without additional kinetic studies. In marine systems, the majority of microplastics are expected to be in the environment for 2–4 years at, or close to sorption equilibrium. Rivers were shown to act as an emission compartment of microplastics ending up in the oceans [13]. Being close to emission sources (e.g., wastewater treatment plants), microplastics in freshwater systems might be more abundant than in the oceans. Shorter residence times of microplastics in freshwater systems could lead to a smaller proportion of microplastics at, or close to sorption equilibrium. Time to reach equilibrium depends on properties of the microplastic material, the pollutant, and characteristics of the water [3]. Sorption capacity is influenced by the properties of the microplastic material itself (e.g., size, polymer type, shape) and of the pollutants (e.g., physicochemical characteristics, hydrophobicity [24]). Fast sorption is expected for pollutants like hydrophobic organic pollutants (HOC) and for small microplastics [3,56]. Sorption equilibrium within 48 h for BPA to PA particles is relatively fast. The equilibrium partition coefficient indicates similar sorption characteristics, like sorption of phenanthrene to polyvinylchloride in seawater with equilibrium sorption within 24 h [26]. Log K_PA,w_ of 3.22 corresponds to a log K_ow_ of BPA of 3.4 measured in an earlier study [57], indicating that hydrophilicity of BPA is a good estimate for sorption capacity of BPA to PA particles. The partition coefficient (10^3^ L kg^−1^) is within the range of HOC sorption to microplastics in seawater (10^2^–10^7^ L kg^−1^; [58]). Competitive sorption by other pollutants and leaching additives influence sorption behavior as well, but were not analyzed in this study [59].

### 4.2. Exposure Experiments with Mixtures of BPA and PA Particles

Besides sorption behavior, also organism dependent factors need to be considered for the potential vector effect of microplastics, i.e., uptake of microplastics, as well as conditions and processes within the organism. Two possible uptake pathways for BPA were included in the experiments: direct uptake by BPA dissolved in water, and vector-based uptake by ingestion of PA particles loaded with BPA. Microplastics tested in most studies were the only uptake pathway for the pollutants [34]. Other media, e.g., water, prey and detritus, which were shown to also hold a fraction of the pollutants, have not been included [3]. Since daphnids are organisms living in the water column, an important uptake pathway of nutrients, but also pollutants, is water. This is why water was selected as an additional uptake pathway for BPA in the simplified exposure scenario of this study. Non-suspended microplastics (e.g., aggregated at the water surface or settled) were discussed to reduce interactions of test organisms, leading to reduced effects of microplastic associated pollutants [54]. In this study, grazing by daphnids on settled PA particles from the bottom of the test beakers was observed. We consider high ingestion rates of microplastics by the daphnids, because intestines were observed to be filled up with PA particles within the first 24 h until the end of the test. Grazing with high uptake rates ensured availability of PA particles, which could then potentially act as vector for BPA. Daphnids were also able to egest PA particles. Quantification of exact ingestion and egestion rates was beyond the focus of this study, but would allow getting a deeper understanding of the processes within the daphnids. The scope of furthermore refined studies could be to analyze, e.g., if the time span of the microplastic particles within the daphnids is related to sorption and desorption kinetics.

Immobilization of daphnids was analyzed as an experimental endpoint to directly determine the influence of microplastics on pollutant toxicity. The pairwise comparison of immobilization rates with the same nominal concentration of BPA directly compares scenarios with the same overall mass of BPA without taking the distribution of BPA into account. The increase of immobilization rates in a dose-dependent manner for nominal concentrations follows the known acute toxicity pattern for BPA. The same dose-dependent pattern for BPA, in combination with microplastics but with overall lower immobilization rates, shows that the presence of PA particles reduced immobilization in daphnids.

Water seems to be the most bioavailable fraction, as hypothesized. Analytical measurements showed that decreased immobilization for BPA in combination with microplastics was associated with lower BPA concentrations in water compared to BPA alone. Sorption of BPA to PA particles led to lower actual concentrations of BPA in water, already during preparation of test solutions. Sorption of PCB to microplastics (PE, 10–180 μm) with dilution of PCB in water was shown to compensate possible vector effects, contributing to bioaccumulation of PCB in lugworms [38]. That sorption of phenanthrene to microplastics (unplasticized polyvinyl chloride, 200–250 μm) can lead to reduced effect rates of the compound was shown by biomarker activity in zebra fish larvae [60]. Vector-based uptake by ingestion was excluded, because larvae did not develop mouthparts yet. Larvae interacted with microplastics only by dermal contact. Even though PA particles loaded with a fraction of the pollutant were ingested by the daphnids, this vector-based uptake of the pollutant does not seem to compensate reduced uptake from water. The overall effect of the pollutant seems to depend mainly on the amount of dissolved pollutant in water, if sorption equilibrium is assumed.

While comparing the same nominal concentrations of BPA clearly showed a reduction of immobilization in the presence of PA particles, it cannot be excluded that a fraction of BPA causing immobilization was associated to PA particles. How much the single fractions (water, microplastics) contributed to the overall effect of BPA can be addressed by comparing EC_50_ values. EC_50_ values were calculated with immobilization rates and actual concentrations of BPA measured by HPLC in water. If only BPA dissolved in water is determining the rate of immobilization, EC_50_ values of BPA alone, and BPA in combination with microplastics, can be expected to be in the same range. A lower EC_50_ for BPA in combination with microplastics would indicate higher sensitivity of daphnids to BPA if PA particles are present. Although the EC_50_ for BPA in combination with microplastics is lower than for BPA alone, the overlapping confidence intervals indicate no significant difference. Thus, we consider the contribution of PA particles as a source for BPA to the overall immobilization rate to be negligibly small.

Tissue concentrations of pollutants were measured in most studies to analyze the potential vector effect of microplastics. While we did not measure internal conditions, including tissue concentrations, calculations based on physicochemistry give an indication for the overall uptake of BPA. Calculations on the mass distribution of BPA indicate that not only less BPA is partitioning into water, but also, less BPA is distributed into the daphnids. Lower calculated body burden with BPA corresponds to observed lower immobilization rates. In adult zebra fish tissue, concentrations of silver (Ag) were reduced, if Ag could sorb to microplastics during 96 h incubation prior to exposure [54]. In another study, whole body concentrations of phenanthrene in daphnids were not different between treatments with microplastics or phenanthrene alone [22]. Only nanoplastics enhanced phenanthrene uptake in this study, which stresses the bigger vector potential of plastics below micro scale.

Different factors have been discussed to influence desorption of pollutants within organisms between ingestion and egestion of microplastics, i.e., pre-exposure with pollutants, biological conditions, and processes. The concentration gradient in this study was not influenced by pre-experimental BPA burden in daphnids, because the daphnids have not been exposed to BPA before exposure. When organisms have already accumulated pollutants in their body, remobilization of the pollutant from microplastics can be expected to be lower because of smaller concentration gradients. Ingestion of relatively clean microplastics is discussed to reduce pollutant burden in organisms, if partition coefficients are higher for the plastic material [61]. Three week exposure of lobsters with microplastics loaded with PCBs and incorporated in food had no effect on PCB concentrations in tail tissue [43]. The lobsters had been pre-exposed to PCBs prior to experiments in the environment, which resulted in a smaller concentration gradient compared to clean organisms. After a depuration phase of one week with ingestion of clean microplastics, PCB concentrations were the same, indicating no cleaning effect of microplastics.

Based on experimental evidence, physiological conditions in the gut, like gut surfactants, pH, and temperature, were discussed to enhance remobilization of absorbed pollutants on microplastics [25]. Faster desorption rates were found only for warm-blooded organisms. Model-based studies on marine organisms hypothesize that desorption of organic pollutants from microplastics is negligible, even if physiological factors are included [37]. Interactions between organismal tissue and ingested microplastics loaded with pollutants also depend on gut passage time. Higher remobilization rates of pollutants from microplastics can be expected for longer gut passage times. Gut passage time in daphnids for food particles is relatively short, with egestion within minutes [62]. Thus, remobilization from loaded microplastics which pass through the digestive system might be limited. Smaller microplastics which are able to pass tissue or even cell barriers, might be of more importance for acting as vectors, while bigger microplastics can be egested more easily [22]. In addition to microplastics in the intestinal tract of daphnids, translocation of PA particles within the body, like observed for 1 μm microplastics [21], cannot be excluded.

Besides tissue concentration, also the location of a pollutant within the body and depuration was shown to be influenced by microplastics. A bigger proportion of Ag was found to be located in the intestines in the presence of microplastics in zebra fish, although overall Ag concentration was lower compared to exposure without microplastics [54]. Gut content was not separated from organismal tissue for analysis. This is why the higher proportion could be due to microplastics still carrying Ag, rather than higher concentrations of Ag in organismal tissue.

Similar to the results of this study, the role of microplastics as vector seemed small as soon as other uptake pathways than microplastics were included in recent studies. In a sediment-living marine worm, PCB uptake from microplastics was lower than from sediment [41]. Gut solubilization potential was relatively low compared to natural material, i.e., wood and biochar, indicating the limited role of microplastics in pollutant transfer. In marine mussels, a mixture of fluoranthene (Flu), microplastics (PS, mix of 2 and 6 μm), and food algae did not change the concentration of Flu in digestive glands after seven days, compared to Flu and algae without microplastics [42]. By incubating Flu with microplastics and food algae prior to exposure, different uptake pathways were included in this study (water, microplastics, algae). A fraction of Flu which was held by algae was transferred to microplastics during incubation, due to the higher partition coefficient. During a seven day period without any exposure, depuration was lower if mussels had been receiving mixtures, including microplastics, beforehand. Negative effects on detoxification and impairment of the filter activity were discussed as reasons. Also, remaining microplastics loaded with Flu could not be excluded. While concentrations of a pollutant within the organism indicate uptake associated to microplastics, it is necessary to also analyze specific effects of the pollutant on the organism. Even if pollutants desorb from microplastics, a negative effect only manifests if the pollutant reaches the target tissue. Tissue concentrations of a pollutant, especially the whole body burden, do not necessarily reflect the extent of a net pollutant effect. Studies including effects of pollutants, e.g., toxicity, can help to identify the actual influence of microplastics on organisms. Interestingly, toxic effects of Flu on mussels were enhanced for treatments including microplastics, although concentrations of Flu in tissue were not different [42]. More histopathlogical damage and higher activity of antioxidant markers were found.

Besides studies showing low evidence for microplastics as carriers for pollutants, there are also reports about enhanced pollutant body burden and negative effects after ingestion of loaded microplastic [32,33,34]. Different conclusions have been made about the role of microplastics as vector, because of different outcomes of experimental studies. Experimental approaches in these studies differed a lot, but re-evaluation including equilibrium sorption showed that most studies indicate no, or only low relevance for microplastics as pollutant vector [3]. In a recent study, model and experimental approaches were combined to analyze the vector effect of microplastics (PE) for PCB on marine lugworms [38]. Uptake fluxes from all exposure pathways were quantified to comply with environmental relevant exposure conditions. Experimental and model approaches both go along with the general results of our study, that the role of microplastics as vector for organic pollutants is small.

## 5. Conclusions

The exposure scenario in this study addressed selected requirements for an environmentally relevant exposure, i.e., sorption equilibrium and water, as an additional uptake pathway in addition to microplastics. All BPA concentrations used in this experiment greatly exceed concentrations of BPA detected in rivers and lakes, e.g., with a maximum of 16 ng L^−1^ in WWTP effluent [63]. The concentration of the PA particles is also above expected values in freshwater environments [8,9]. Nevertheless, high concentrations of BPA and PA particles, exposure of clean daphnids, and high uptake rates of PA particles, created a scenario in favor of high sensitivity to detect the potential vector effect of PA particles in general. Supporting model-based studies, a vector effect as shown in other experimental studies only plays a minor role when experiments are carried out under more environmentally relevant conditions, and under the assumption of sorption equilibrium (e.g., [3]). These findings help to systematically identify how freshwater organisms are harmed by pollution of chemicals and microplastics, and support a more mechanism-based risk assessment. Further experimental studies could analyze, e.g., how higher sorption capacity of microplastics and other additional uptake pathways might influence a potential vector effect of microplastics, and if other organisms respond in a similar way. Natural particulate matter seems to make a big proportion of particles in aquatic systems. Thus, analyzing their potential vector function in comparison to microplastics could help to set the role of microplastics as pollutant vectors into perspective.

## Figures and Tables

**Table 1:** Mass distribution of bisphenol A (BPA) for all relevant compartments in the test system calculated for BPA alone and BPA in combination with microplastics (MP) (treatment BPA + MP).

Compartment	Mass Distribution of BPA (%)	
	BPA alone	BPA + MP
water	99.72	74.85
organisms	0.28	0.21
PA-particles	-	24.94

PA: polyamide.

## Supplementary Materials

The following are available online at http://www.mdpi.com/1660-4601/15/2/280/s1, Table S1: Measured concentrations (mg L^−1^) of bisphenol A (BPA) in water after 0, 6, 24, 48 and 72 h of shaking with and without polyamide particles (microplastics, MP) (*n* = 2), Table S2: Measured bisphenol A (BPA) concentrations (mg L^−1^) in water in test vessels at the beginning and the end of exposure experiments (mean ± SD, *n* = 5).
