# Supplementary material for: Microplastics Reduce Short-Term Effects of Environmental Contaminants. Part I: Effects of Bisphenol A on Freshwater Zooplankton Are Lower in Presence of Polyamide Particles
Source: Int J Environ Res Public Health. 2018 Feb 6;15(2):280. doi: 10.3390/ijerph15020280 (PMC5858349; doi:10.3390/ijerph15020280)
Supplement: Supplementary file 1 [file ijerph-15-00280-s001.pdf]

International Journal of
Environmental Research and Public Health

Microplastics Reduce Short-Term Effects of Environmental Contaminants

Part I: Effects of Bisphenol A on Freshwater Zooplankton Are Lower in Presence of Polyamide Particles

Saskia Rehse [1,2,*], Werner Kloas [1,3] and Christiane Zarfl [2]

1 Leibniz-Institute of Freshwater Ecology and Inland Fisheries, Müggelseedamm 310, D-12587 Berlin; werner.kloas@igb-berlin.de
2 Center for Applied Geosciences, Eberhard Karls Universität Tübingen, Hölderlinstraße 12, D-72074 Tübingen; christiane.zarfl@uni-tuebingen.de
3 Department of Endocrinology, Institute of Biology, Humboldt-Universität Berlin, Invalidenstraße 110, D-10115 Berlin
* Correspondence: rehse@igb-berlin.de

Supplementary Material

Preparation of batches with bisphenol A alone and in combination with microplastics

Bisphenol A (BPA) solutions with an initial concentration of 10 mg L^{-1} were prepared by dilution of the stock solution with AdaM medium [1]. To reach a final concentration of 200 mg L^{-1} for polyamide (PA) particles (microplastics, MP), 50 mg of the particles were weighed into 20 mL glass flasks. After adding 20 mL of BPA solution and shaking, the mixture was decanted into 500 mL glass bottles. The glass flasks were rinsed three times with BPA solution to ensure complete transfer of particles. Glass bottles were then filled with BPA solution to a total volume of 250 mL resulting in a mixture of BPA and PA particles (BPA + MP). As a control, a treatment with only BPA solution was analysed in parallel (BPA alone). Both treatments (BPA + MP, BPA alone) were run in duplicates. The bottles were continuously shaken at 200 rpm to facilitate continuous contact of particles with BPA. The concentrations of solved BPA in water were analyzed after 0, 6, 24, 48, and 72 hours by high performance liquid chromatography (HPLC) with fluorescence detection (Dionex Ultimate 3000 HPLC with Nova-Pak C18 column). For this purpose, we took an equivalent of 1 mL from each bottle and filtered it with a syringe filter (Whatman Spartan HPLC cert. syringe filter, 13 mm diameter, 0.45 µm pore size) to exclude PA particles from the analysis. The filtrate was diluted to reach a nominal concentration of 2 mg L^{-1} and filled in 1.5 mL glass vials, which were then placed into the autosampler of the HPLC. Samples were measured within 24 hours. Results were multiplied by the corresponding dilution factor. HPLC measurement accuracy of 0.1 mg L^{-1} led to increasing inaccuracies of the measured values with increasing concentrations of BPA, due to dilution.

Exposure with single substances for range finding

Stock solutions with PA particles were prepared to facilitate preparation of test solutions. For both, exposure with PA particles and BPA alone, three replicates with five animals each were exposed per concentration as recommended by the Daphnia sp. Acute Immobilization Test for range finding of concentrations [2]. Glass beakers (total volume of 50 mL) were filled with 40 mL ADaM medium spiked with either PA particles or BPA followed by careful transfer of five newborn daphnids not older than 24 hours per beaker. Beakers were covered with glass petri dishes and daphnids exposed at culturing conditions (temperature 20 ± 2 °C, 16:8 light–dark period) for up to 48 hours. Immobilization was the criterion for acute negative effects.

Sorption characteristics and BPA content of PA particles

During the batch experiment addressing the sorption capacity of the PA particles, concentrations of BPA in water were continuously lower with PA than without (Table S1). BPA concentrations in the solution with BPA alone ranged from 9.5–10.0 mg L^{-1}. For BPA in combination with PA the concentration of BPA decreased and stagnated after 48 hours at 7.5 mg L^{-1}.

Table S1. Measured concentrations (mg L^{-1}) of bisphenol A (BPA) in water after 0, 6, 24, 48 and 72 hours of shaking with and without polyamide particles (microplastics, MP) (n = 2).

Time (hours)	Measured BPA Concentration (mg L^{-1} means ± SD)	
	BPA	BPA + MP
0	9.5 ± 0.0	9.0 ± 0.5
6	9.5 ± 0.0	8.0 ± 0.5
24	10.0 ± 0.0	8.0 ± 0.5
48	10.0 ± 0.0	7.5 ± 0.0
72	9.5 ± 0.0	7.5 ± 0.0

SD: standard deviation.

Concentrations of BPA in Water During Exposure

Comparing concentrations of BPA in water in the test beakers after 0 and 48 hours of exposure, all treatments with BPA alone and BPA in combination with PA were stable, with slightly decreasing concentrations with 0.2 mg L^{-1} in average (Table S2). Concentrations for treatments with BPA in combination with PA were lower than in treatments with BPA alone from the beginning of the test.

Table S2. Measured bisphenol A (BPA) concentrations (mg L^{-1}) in water in test vessels at the beginning and the end of exposure experiments (mean ± SD, n = 5).

BPA Nominal (mg L^{-1})	Measured BPA Concentration (mg L^{-1} means ± SD)			
	BPA Alone		BPA + MP	
	0 h	48 h	0 h	48 h
5	5.3 ± 0.0	4.8 ± 0.1	3.7 ± 0.1	3.4 ± 0.1
7.5	7.3 ± 0.3	6.8 ± 0.2	6.0 ± 0.0	5.6 ± 0.0
10	10.2 ± 0.4	9.9 ± 0.2	8.1 ± 0.2	7.5 ± 0.3
12.5	13.0 ± 0.3	13.0 ± 0.3	10.1 ± 0.3	-
15	15.8 ± 0.5	15.8 ± 0.0	12.8 ± 0.0	12.0 ± 0.0

SD: standard deviation.

References

1. Klüttgen, B.; Dülmer, U.; Engels, M.; Ratte, H. ADaM, an artificial freshwater for the culture of zooplankton. *Water. Res.* **1994**, *28*, 743–746.
2. Organization for Economic Co-Operation and Development. *OECD Guidelines for the Testing of Chemicals, Section 2*; Test No. 202: *Daphnia* sp. Acute Immobilisation Test; OECD Publishing: Paris, France, 2004.
